# Examining the Interactive Associations of Cannabis and Alcohol Outlets With Self-harm Injuries in California: A Spatiotemporal Analysis

**DOI:** 10.1097/EDE.0000000000001822

**Published:** 2024-12-16

**Authors:** Rafael Charris, Jennifer Ahern, Dorie E. Apollonio, Victoria Jent, Laurie M. Jacobs, Shelley Jung, Laura A. Schmidt, Paul Gruenewald, Ellicott C. Matthay

**Affiliations:** From the a Center for Opioid Epidemiology and Policy, Division of Epidemiology, Department of Population Health, New York University Grossman School of Medicine, New York, NY; b Division of Epidemiology and Biostatistics, School of Public Health, University of California, Berkeley, CA; c School of Pharmacy, University of California, San Francisco, San Francisco, CA; d Philip R. Lee Institute for Health Policy Studies, School of Medicine, University of California, San Francisco, San Francisco, CA; e Department of Humanities and Social Sciences, School of Medicine, University of California, San Francisco, San Francisco, CA; f Prevention Research Center, Pacific Institute for Research and Evaluation, Berkeley, CA.

**Keywords:** Alcohol, California, Cannabis, Marijuana, Outlet density, Policy, Self-harm, Suicide

## Abstract

**Background:**

Cannabis use and alcohol use are associated with self-harm injuries, but little research has assessed links between recreational cannabis outlet openings on rates of self-harm within communities or the interactions of cannabis outlets with the density of alcohol outlets. We estimated the associations of recreational cannabis outlets, alcohol outlets, and their interaction on rates of fatal and nonfatal self-harm injuries in California, 2017–2019.

**Methods:**

Using California statewide data on recreational cannabis outlets, alcohol outlets, and hospital discharges and deaths due to self-harm injuries, we conducted Bayesian spatiotemporal analyses of quarterly ZIP code-level data over 3 years, accounting for confounders and spatial autocorrelation. Using the model posteriors, we estimated parameters corresponding to hypothetical shifts in outlet densities.

**Results:**

If recreational cannabis outlets had never opened, we estimated that nonfatal self-harm injuries would have been −0.35 per 100,000 lower (95% credible interval [CI]: −1.25, 0.51), while fatal self-harm injuries would have been −0.004 per 100,000 lower (95% CI: −0.26, 0.25). These associations did not depend on alcohol outlet density, but a hypothetical 20% reduction in alcohol outlet densities was associated with fewer self-harm injuries (risk difference per 100,000, nonfatal: −1.59; 95% CI: −2.60, −0.59; fatal: −0.10; 95% CI: −0.37, 0.16). Associations for nonfatal incidents were strongest for people aged 15–34 years, and White and Hispanic people.

**Conclusion:**

We did not find evidence that the introduction of recreational cannabis outlets was associated with self-harm injuries or that cannabis and alcohol outlet densities interact, but alcohol outlet density had a strong association with nonfatal self-harm injuries.

A growing body of research has explored the health impacts of state recreational cannabis policies in the United States.^1^ Most states that legalized recreational cannabis use permit recreational cannabis outlets that offer retail sales of legal cannabis products. Public health experts have proposed that greater availability of cannabis through legal outlets will lead to increases in consumption, as has been observed for alcohol.^2,3^ In fact, research has linked cannabis outlet density to health-related outcomes, including cannabis use, cannabis use disorder, and opioid misuse.^4–9^ One understudied outcome that may be affected by recreational cannabis outlets is self-harm injury, which in 2021 was the second leading cause of death for people aged 10–34 years.

Previous research has documented associations between cannabis use and mental health problems, including schizophrenia, attention-deficit/hyperactivity disorder, depression, anxiety, and suicidality (suicidal ideation, planning, attempts, or death by suicide).^10–13^ One study indicated that cannabis use disorder was associated with a higher risk of nonfatal self-harm injuries in adolescents.^14^ In a study of twins discordant for cannabis use disorder, cannabis dependence was associated with an elevated risk of suicidality and major depressive disorder.^15^ Furthermore, a meta-analysis found that chronic, but not acute, cannabis use was associated with an increased risk of suicidality.^16^ Research using Mendelian randomization, a design that strengthens confounding control through the use of genetic variation as an instrumental variable, estimated a causal effect of cannabis use on the risk of schizophrenia but no evidence of a causal effect on bipolar disorder or attention-deficit/hyperactivity disorder.^11,17–20^

It is important to consider this evidence in light of the current cannabis legalization wave. Specifically, public health research must assess whether cannabis legalization and corresponding increases in legal cannabis availability through outlets have led to increases in cannabis-related harms. Using state- or county-level panel data, one group of studies found no association between medical marijuana legalization and suicide risk.^21–23^ However, another study found a protective association with medical cannabis legalization for men aged 20–39 years and fatal self-harm.^24^ Research on recreational cannabis legalization is also mixed. One study found no association with hospitalizations for self-harm, whereas two others found evidence of increased risk of suicide after legalization for people aged 14–16 and 15–24 years.^25–27^

One potential explanation for these inconsistent findings may be differences in the role of alcohol contexts across studies. Alcohol has a strong relationship to self-harm.^28,29^ In places with high densities of alcohol outlets (and hence greater availability and consumption of alcohol), self-harm risk may already be elevated, and the introduction of cannabis outlets may matter less for cannabis use-related risk.^30,31^ Consequently, studies on the links between cannabis outlets and self-harm based in settings with high densities of alcohol outlets may produce different findings from studies in low-alcohol outlet density settings. If verified, this pattern would have important implications for the geographic patterning of the public health impacts of cannabis legalization.

To our knowledge, no research has taken a geospatial approach to examine the association of recreational cannabis outlets with rates of self-harm injuries or how alcohol outlet density may modify this association. Prior research has focused exclusively on state- or county-level associations of cannabis legalization policies with self-harm outcomes using differences-in-differences or interrupted time series. To fill this gap, we used Bayesian spatiotemporal analyses to estimate the associations of recreational cannabis outlets, alcohol outlets, and their interaction on rates of fatal and nonfatal self-harm injuries across California. Given the mixed evidence to date, we hypothesized that the combined association of cannabis and alcohol outlets with self-harm rates will be less than expected based on associations for either substance alone (i.e., negative interaction). Because fatal and nonfatal self-harm injuries have distinct risk profiles (fatal self-harm injuries are most common among older men, whereas nonfatal self-harm injuries are most common among younger women), we examine fatal and nonfatal injuries separately.^32,33^ To understand how cannabis and alcohol outlets relate to disparities, we also considered effect measure modification by age, gender, and race–ethnicity.

## METHODS

### Overall Approach

We compiled and analyzed spatial panel data at the level of the ZIP code Census Tabulation Area (ZCTA) and quarter from 2017 to 2019 (1769 ZCTAs over 11 quarters [3-month periods] or 21,228 total space-time units). This study period spans the date when the recreational cannabis outlets first started sales on 1 January 2018. Note that this analysis estimates the average treatment effect on the treated, and therefore, the positivity assumption requires that all covariate subgroups that have treated units also have untreated units, but the reverse is not required. Our design leverages within-ZCTA comparisons before and after the introduction of recreational cannabis outlets to strengthen causal inferences. We used ZIP codes because they are the most granular geographic unit available in the hospital discharge data. ZCTAs are the US Census Bureau’s areal representations of US Postal Service’s ZIP code delivery routes, typically contain about 20,000 people, and correspond to communities or neighborhoods in urban areas and larger regions in rural areas. The Institutional Review Boards of New York University Grossman School of Medicine, California Health and Human Services Agency, and the University of California Berkeley approved this study.

### Alcohol and Cannabis Outlet Data and Exposure Measures

We gathered yearly data on active alcohol licenses from 2017 to 2019 from the California Department of Alcoholic Beverage Control. These listings, known for their accuracy, are frequently utilized in studies of alcohol-related harms.^2,30,34^ Alcohol outlet density for each ZCTA-quarter was defined as the number of establishments selling alcohol per 100,000 inhabitants.

Data on cannabis outlets were gathered quarterly from 2017 to 2019 from Weedmaps, an online cannabis retail locator commonly used in cannabis research. A prior validation study revealed that Weedmaps provided the most up-to-date and comprehensive information on cannabis outlets compared with official licenses or other databases.^35^ Given that ZCTAs seldom contained more than one cannabis outlet, our measure of cannabis outlet exposure was a binary indicator of the existence or nonexistence of any recreational cannabis storefronts in the specified ZCTA and quarter. We focused on recreational outlets rather than medical ones due to the scarcity of medical-only outlets postrecreational legalization. Recreational outlets encompassed both newly established outlets and those that transitioned from medical to recreational. We prioritized storefront outlets (which offer in-person sales) over home delivery retailers, as no established methods for measuring access through delivery exist.^36^ For a more detailed discussion, refer to the “Cannabis outlet measurement” section in eAppendix; http://links.lww.com/EDE/C206.

We geocoded the addresses of both alcohol and cannabis outlets to ZCTAs using Geocodio, with a success rate of over 99%

### Self-harm Injury Data and Outcome Measures

We obtained statewide emergency department and inpatient hospital discharge records from the California Department of Health Care Access and Information and death records from the California Office of Vital Statistics from 2017 to 2019. From these records, we identified cases of fatal and nonfatal self-harm injuries using the external cause of injury code (see eTable 1; http://links.lww.com/EDE/C206 for International Classification of Diseases codes). Discharge records included the residential ZIP codes of the injured individuals which we translated to ZCTAs using an established crosswalk (see Supplemental Digital Content; http://links.lww.com/EDE/C206). In California, injury coding in hospital discharge records is compulsory, continuously monitored for quality, and deemed to be 100% complete.^37^ The validity of self-harm International Classification of Diseases codes in hospital and death records is generally accepted.^38–40^ The primary outcome measures were rates of fatal and nonfatal self-harm injuries by ZCTA and quarter, using population denominators from the American Community Survey.

### Confounder Data and Measures

The adjustment set included confounders that are known to influence the density or locations of alcohol and cannabis outlets, as well as the risk of self-harm.^41^ These confounders encompassed demographic composition and socioeconomic factors (race–ethnicity, poverty, unemployment, education, income inequality, population density, median age, renters, and veterans). We also incorporated continuous quarter fixed effects (1–12) to control for temporal patterns applicable to all ZCTAs, such as statewide policy modifications and seasonal variation in self-harm. Detailed information on each variable’s data sources and procedures can be found in eTable 2; http://links.lww.com/EDE/C206.

### Statistical Analysis

Of 1769 ZCTAs, we omitted 45 that had no residential populations and another 49 missing key covariates. The final dataset included 1675 ZCTAs across 11 quarters. We modeled self-harm injuries as a Poisson process and applied Bayesian spatiotemporal regression to measure the relationships of cannabis outlets, alcohol outlet density, and their interaction with self-harm injuries. This method employs conditional autoregressive random effects to address spatial autocorrelation in self-harm rates across neighboring ZCTAs, which could otherwise bias point estimates and standard errors.

Our model specification was as follows:


$$\begin{array}{l} Y_{it}|\mu_{it}∼Poisson\left(E_{it}\exp\left(\mu_{it}\right)\right) \end{array}$$

$$\begin{array}{l} \mu_{it}=\beta_{0}+\beta_{1}AOD_{it}+\beta_{2}CO_{it}+\beta_{3}AOD_{it}CO_{it}+\beta_{4}X_{it}+\alpha_{t}+\omega_{i}+\psi_{i} \end{array}$$

where $Y_{it}$ was the number of self-harm injuries in ZCTA i at time t, $E_{it}$ was the expected number of injuries assuming a distribution proportional to population ($E_{it}=P_{it}\frac{\sum Y_{it}}{\sum ,P_{it}}$ where $P_{it}$ is the population of the *i*th ZCTA at time *t*, analogous to including a population offset to model rates), $\exp\left(\mu_{it}\right)$ was the relative risk of a self-harm injury, $\beta_{0}$ was the intercept, $AOD_{it}$ was alcohol outlet density, $CO_{it}$ was a binary indicator of the presence of any cannabis outlets, $exp(\beta_{1}),\exp\left(\beta_{2}\right)$, and $\exp\left(\beta_{3}\right)$ were the estimated relative risks indicating the associations of alcohol outlet density, cannabis outlets, and their interaction with self-harm injuries, respectively, $X_{it}$ was a matrix of confounders, $\beta_{4}$ was a vector of coefficients for each confounder, $\alpha_{t}$ were indicator variables representing the year-quarter (t = 1, …, 12), $\omega_{i}$ were ZCTA random effects intercepts assuming independence of units (spatially unstructured), and $\psi_{i}$ were spatially structured random effects intercepts allowing self-harm counts for neighboring ZCTAs to be dependent, with neighboring ZCTAs defined as those that shared a border (i.e., “queen adjacencies”). We removed model terms that did not improve model fit, where we defined improved model fit as a 5-unit reduction in the Watanabe-Akaike Information Criterion.^42,43^

Following previous research, we estimated the coefficients using Integrated Nested Laplace Approximation using default priors.^44–47^ We used a reparametrization of the Besag-York-Mollié model to assess spatial autocorrelation. The reparametrization (Besag-York-Mollié-2) better handles noncontiguous geographic units or “islands.”^48,49^ We did not use a misalignment model because ZCTAs were defined consistently over the study period. To aid interpretation, we used g-computation on samples of the posterior distributions from the fitted models to calculate risk differences (RDs) comparing the outcomes under the observed distributions of cannabis and alcohol outlets versus the predicted outcomes under hypothetical reductions in cannabis outlets and alcohol outlet density.^49^ We estimated how self-harm injuries would differ from observed if (a) cannabis outlets never opened (i.e., reduced by 100%), (b) if alcohol outlet density were reduced by X% in each ZCTA-quarter with X ranging from 10% to 90%, and (c) both changes occurred at the same time. We assessed interaction on the additive scale by assessing whether the RD for the hypothetical reduction in both cannabis and alcohol outlets was bigger than the sum of the RDs for reductions in cannabis and alcohol outlets independently. We report estimates as median risk differences per 100,000 with 95% credible intervals. The code is provided in the Supplemental Digital Content; http://links.lww.com/EDE/C206.

### Secondary and Sensitivity Analyses

In secondary analyses, we examined associations by age group, sex, and race–ethnicity. We also added spatially lagged exposure terms to test the impacts of the density of alcohol outlets and the presence of cannabis outlets in neighboring ZCTAs on outcomes in the index ZCTA.

We conducted several sensitivity tests. First, we added a quadratic alcohol outlet density term to test whether there was a nonlinear relationship with the outcome. Second, we added a term to control for the baseline density level of medical dispensaries to account for the historical medical market. Third, to reduce the impact of outliers, we excluded ZCTAs with fewer than 500 people at any time point. Fourth, we ran models with ZCTA-level random slopes to allow for each ZCTA to have a unique trend in the self-harm injury rate. Fifth, we tested models using ZCTA fixed effects instead of random effects. These models do not adjust for spatial autocorrelation but better control for time-invariant ZCTA-level confounders (e.g. geography, climate, political orientation). Finally, because the impacts of alcohol outlet density may vary by type of alcohol outlet, we added terms representing the proportion of alcohol outlets that were (a) bars/pubs, and (b) stores offering alcohol for off-premises consumption. We incorporated these terms as proportions rather than densities to address numerical problems that arise when assessing exposures that are components of a total. Finally, we ran the model separately for urban and rural ZCTAs, classifying them based on the rural-urban commuting areas.^50,51^

## RESULTS

**Figure 1.**
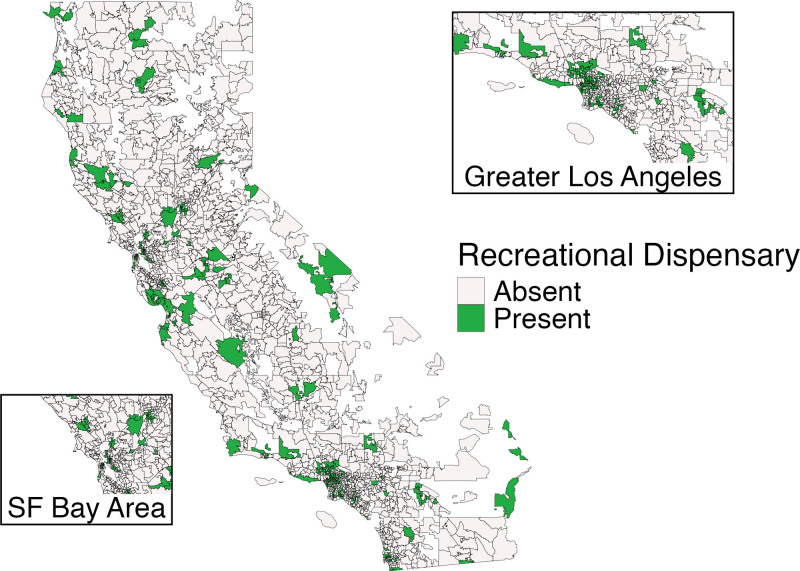
ZIP code census tabulation areas with one or more storefront recreational cannabis outlets, California, 2019.

**Figure 2.**
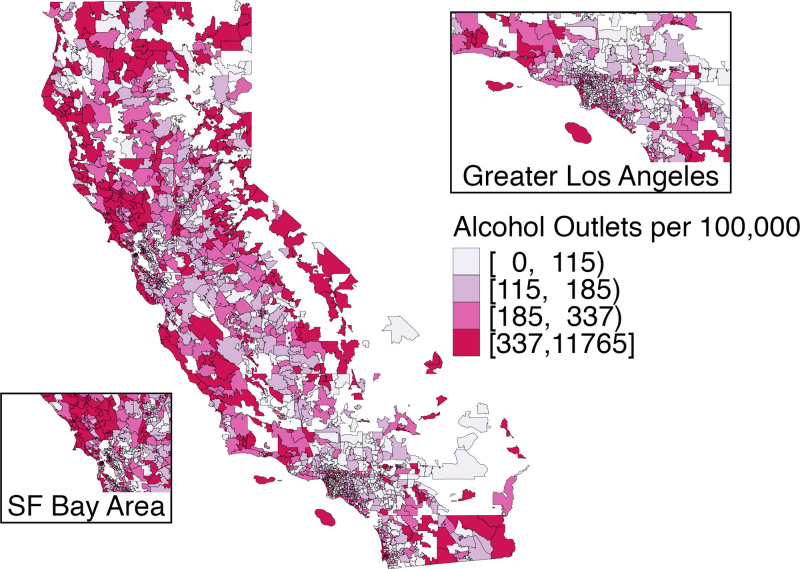
Alcohol outlet density by ZIP code census tabulation areas California, 2019.

Of 1675 ZCTAs, 234 had at least one recreational cannabis outlet by the end of 2019 (Figure 1). Cannabis outlets were present primarily in urban areas. Alcohol outlet density also varied widely across the state (Figure 2).

The Table describes the characteristics of the study units (ZCTA-quarters) by whether or not they had any recreational cannabis outlets. The distribution of age, sex, education, poverty, and density of alcohol outlets was similar for both groups. However, ZCTA-quarters with cannabis outlets had higher population densities, more renters, more Hispanic residents, and fewer White residents on average compared to ZCTA-quarters without cannabis outlets.

**Table.** Characteristics of ZCTA-Quarters With and Without Storefront Recreational Cannabis Outlets, California, 2017–2019

Variable	Level	Storefront Recreational Cannabis Outlets	
		Absent	Present
		N = 29,006	N = 1,141
		Median (First Quartile, Third Quartile)	
Age (yrs)	<15	18% (14%, 22%)	17% (13%, 21%)
	15–34	12% (9%, 14%)	12% (9%, 15%)
	35–54	26% (23%, 28%)	26% (24%, 28%)
	≥55	23% (18%, 30%)	20% (17%, 24%)
Sex	% Female	50% (48%, 52%)	50% (48%, 51%)
Race/ethnicity	American Indian/AK Native	0% (0%, 1%)	0% (0%, 0%)
	Asian/Pacific Islander	4% (1%, 12%)	7% (3%, 15%)
	Black	2% (0%, 4%)	3% (1%, 7%)
	Hispanic	22% (11%, 44%)	30% (16%, 54%)
	Other race	0% (0%, 0%)	0% (0%, 0%)
	White	54% (29%, 75%)	41% (20%, 64%)
	Two or more races	3% (1%, 4%)	3% (2%, 5%)
Educational attainment	High school or GED	22% (15%, 28%)	21% (13%, 26%)
	At least some college	64% (50%, 77%)	63% (49%, 79%)
	At least bachelor’s	27% (16%, 44%)	31% (18%, 50%)
	Master’s, doctoral, or professional degree	9% (4%, 17%)	10% (5%, 19%)
Families in poverty		7% (4%, 14%)	9% (5%, 14%)
Veterans		8% (6%, 10%)	9% (8%, 12%)
Unemployment		6% (4%, 9%)	6% (5%, 8%)
No health insurance		8% (5%, 12%)	8% (5%, 12%)
Gini coefficient		0.43 (0.39, 0.46)	0.45 (0.41, 0.48)
Avg household size		3 (2, 3)	3 (2, 3)
Renters		36% (25%, 50%)	48% (36%, 62%)
Population density (per 10 sq km)		2 (0, 18)	16 (2, 38)
Alcohol outlets	Density per 100,000 people	181 (110, 326)	184 (119, 343)
	% on-premise restaurants	50% (31%, 64%)	51% (39%, 64%)
	% on-premise bar/pub	5% (0%, 10%)	8% (5%, 12%)
	% off-premise	42% (27%, 57%)	41% (28%, 55%)

All race groups are non-Hispanic, except Hispanic which includes people of any race.

eTable 6; http://links.lww.com/EDE/C206 shows the average rate of fatal and nonfatal self-harm injuries during the study period.

### Nonfatal Self-harm Injuries

The model’s hyperparameters indicate how much of the variation in self-harm rates is explained by spatial autocorrelation. We found that the spatially structured random intercepts explained 68% of the marginal variance in the ZCTA random intercepts (95% credible interval [CI]: 56%, 78%) (eTable 3; http://links.lww.com/EDE/C206). This means that more than half of the variation in the outcome is explained by the spatial patterning of the data, even after controlling for all the covariates (eTable 2; http://links.lww.com/EDE/C206).

**Figure 3.**
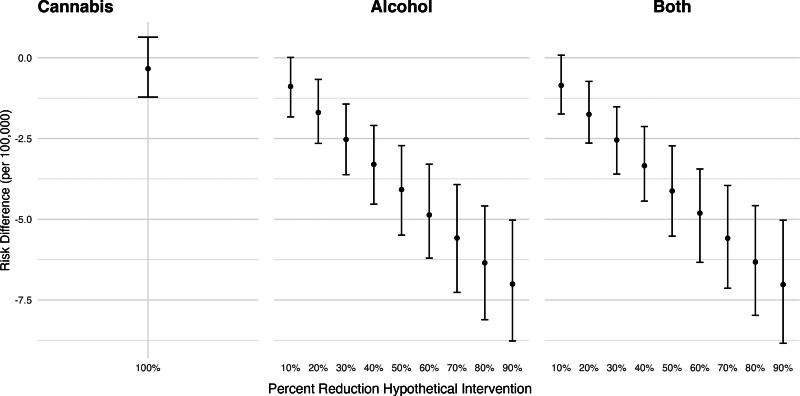
Adjusted risk difference in nonfatal self-harm injuries under hypothetical reductions in alcohol and cannabis outlet densities, California, 2017–2019. Estimated differences in the risk of nonfatal self-harm injuries corresponding to hypothetical reductions in alcohol and cannabis outlet densities, calculated using the posterior distributions of the fitted models. We estimated the change in nonfatal self-harm risk if storefront recreational cannabis outlets had never opened (i.e., setting cannabis outlets to 0 throughout) if alcohol outlet density were X% lower in each ZCTA-quarter where X ranged from 10% to 90%, and if both shifts occurred simultaneously. Estimates are reported as marginal posterior median risk differences per 100,000 with 95% credible intervals.

Figure 3 shows the estimated risk differences in nonfatal self-harm injuries corresponding to hypothetical shifts in cannabis and alcohol outlet densities. We found little difference in nonfatal self-harm injuries if recreational cannabis outlets were never introduced (RD per 100,000: −0.35; 95% CI: −1.25, 0.51). However, we found that reducing alcohol outlet densities in all ZCTAs was associated with a lower risk of nonfatal self-harm injuries. For example, a 20% reduction in alcohol outlet densities was associated with a decrease of 1.59 per 100,000 in the risk of nonfatal self-harm (95% CI: −2.6, −0.59). We found no evidence of interaction between cannabis and alcohol outlets, as the coefficients for the combined shift were of the magnitude expected based on shifting each exposure alone.

**Figure 4.**
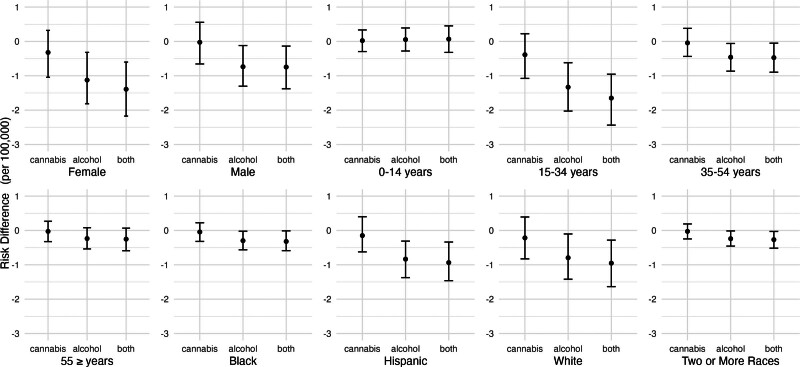
Adjusted risk difference in nonfatal self-harm injuries under hypothetical reductions in alcohol and cannabis outlet densities by population subgroup, California, 2017–2019. Estimated differences in the risk of nonfatal self-harm incidents corresponding to hypothetical shifts in alcohol and cannabis outlet densities, calculated using the posterior distributions of the fitted models. The “cannabis” shift refers to eliminating all cannabis outlets. The “alcohol” shift refers to reducing alcohol outlet density in each ZCTA-quarter by 20%. “Both” refers to the combination of “cannabis” and “alcohol” shifts simultaneously. Estimates are reported as marginal posterior median risk differences per 100,000 with 95% credible intervals. Models for the non-Hispanic Asian/Pacific Islander group did not converge.

Figure 4 shows the estimated risk differences for nonfatal self-harm injuries, by population subgroup, corresponding to eliminating cannabis outlets, reducing alcohol outlet densities by 20%, or both. Associations were most pronounced for people aged 15–34 years, women, non-Hispanic White people, and Hispanic people. Qualitatively speaking, the reduction associated with alcohol outlets was bigger for 15–34 year olds than the other age groups, but our analysis did not formally test whether these coefficients were statistically different.

### Fatal Self-harm Injuries

eTable 3; http://links.lww.com/EDE/C206 presents the estimated hyperparameters for the adjusted spatiotemporal models for fatal self-harm in the overall population. Similar to nonfatal self-harm, there was substantial spatial autocorrelation: 62% of the marginal variance in the ZCTA random intercepts was explained by the spatially structured random intercepts (as opposed to the spatially unstructured random intercepts) (95% CI: 33%, 86%).

**Figure 5.**
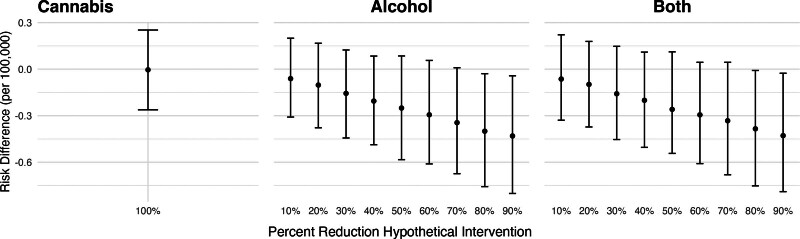
Adjusted risk difference in fatal self-harm injuries under hypothetical reductions in alcohol and cannabis outlet densities, California, 2017–2019. Estimated differences in the risk of fatal self-harm injuries corresponding to hypothetical shifts in alcohol and cannabis outlet densities, calculated using the posterior distributions of the fitted models. We estimated the change in self-harm risk if storefront recreational cannabis outlets had never opened (i.e., setting cannabis outlets to 0 throughout), if alcohol outlet density were X% lower in each ZCTA quarter where X ranged from 10% to 90%, and if both shifts occurred simultaneously. Estimates are reported as marginal posterior median risk differences per 100,000 with 95% credible intervals.

Figure 5 shows the estimated risk differences in fatal self-harm injuries corresponding to hypothetical shifts in cannabis and alcohol outlet densities. Eliminating recreational cannabis outlets was not associated with a change in fatal self-harm injuries (RD per 100,000: 95% CI: −0.26, 0.25). For hypothetical reductions in alcohol outlet densities, the direction of the relationship was similar to that for nonfatal self-harm injuries, but the estimates were smaller in magnitude and less precise. For example, a 20% reduction in alcohol outlet densities was associated with 0.10 fewer fatal self-harm injuries per 100,000 inhabitants (95% CI: −0.37, 0.16).

**Figure 6.**
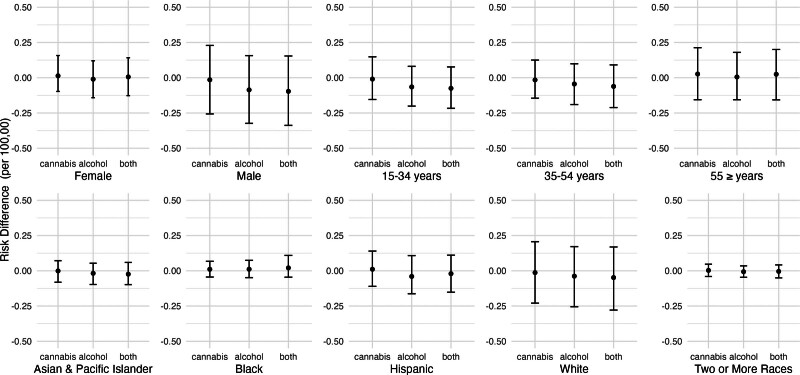
Adjusted risk difference in fatal self-harm injuries under hypothetical reductions in alcohol and cannabis outlet densities, California, 2017–2019. Estimated differences in the risk of fatal self-harm injuries corresponding to hypothetical shifts in alcohol and cannabis outlet densities, calculated using the posterior distributions of the fitted models. We estimated the change in self-harm risk if storefront recreational cannabis outlets had never opened (i.e., setting cannabis outlets to 0 throughout), if alcohol outlet density were X% lower in each ZCTA quarter where X ranged from 10% to 90%, and if both shifts occurred simultaneously. Estimates are reported as marginal posterior median risk differences per 100,000 with 95% credible intervals.

Figure 6 shows the estimated risk differences for fatal self-harm injuries, by population subgroup, corresponding to eliminating cannabis outlets, reducing alcohol outlet densities by 20%, or both. Associations did not vary greatly across groups but were most pronounced for men and people aged 15–34 years old. Estimates were also less precise for men, White people, and people aged 55 years or more. Qualitatively, these coefficients were not different from each other as they were all very close to zero, but our analysis did not test whether these were statistically different.

### Secondary and Sensitivity Analyses for Nonfatal and Fatal Self-harm Injuries

As eTable 5; http://links.lww.com/EDE/C206 shows, the results of the sensitivity analyses were aligned with those of the main analysis, and in eTable 4; http://links.lww.com/EDE/C206, we present a comparison of model fit for secondary and sensitivity analyses. The results suggest that our findings are driven primarily by associations in urban areas: In urban ZCTAs, there is evidence of a decrease in nonfatal self-harm rates associated with a hypothetical reduction in alcohol outlets but not cannabis outlets. In rural ZCTAs, there is no evidence suggesting a reduction in nonfatal self-harm rates after a decrease in cannabis outlets or alcohol outlet density. Incorporating terms for different types of alcohol establishments, spatially lagged terms, including baseline medical dispensaries, adding ZCTAs fixed effects, or a quadratic alcohol outlet density term did not improve the model fit. Excluding ZCTAs with very small populations and adding ZCTA-level random slopes improved model fit.

For fatal self-harm, we observed a similar pattern of findings for fatal self-harm in urban ZCTAs, but the credible intervals were wide, and they all included zero. Models for fatal self-harm in rural ZCTAs did not converge, probably because of the low counts of injuries and spatial sparsity of rural ZCTAs. The only sensitivity test that improved model fit was removing ZCTAs with very small populations. Finally, we combined results for both fatal and nonfatal self-harm incidents as a sensibility analysis. These results were in the same direction as both fatal and nonfatal outcomes, but they did not improve the model fit with respect to any of the two main models.

## DISCUSSION

We analyzed how rates of self-harm injuries in California ZCTAs changed following the introduction of recreational cannabis outlets and in relation to varying densities of alcohol outlets. We observed no meaningful change in fatal or nonfatal self-harm associated with recreational cannabis outlets in the first 2 years of recreational cannabis sales, nor did we find evidence of interaction between cannabis and alcohol outlets. However, a hypothetical reduction in alcohol outlet densities was associated with lower rates of fatal and nonfatal self-harm injuries. This association differed by population subgroup, with the largest estimated reductions for Hispanic people, White people, women, and people aged 15–34 years.

Our observation of no association between self-harm rates and recreational cannabis outlets (and no interaction with alcohol outlets) may have several explanations. First, cannabis use may not actually affect the risk of self-harm. While multiple cohort studies have documented correlations between cannabis use and self-harm, these studies may be confounded by other individual-level risk factors, such as adverse childhood experiences.^52–56^ In contrast, our study may provide stronger confounding control by leveraging exogenous variation in cannabis use induced by cannabis outlet openings. Importantly, the differences in the results between these cohort studies and ours might be due to this study being ecological. It is important to notice that these null results do not preclude the possibility of individual-level effects, but we studied population-level effects only.

Alternatively, recreational cannabis outlets might increase the risk of self-harm injuries, but over longer time periods than those under study. In this study, we observed only the first 2 years of recreational cannabis sales, ending with a total of 488 operational outlets. Future research capturing longer follow-up periods after more recreational outlets appeared would be informative. With more outlets or detailed sales data, future research might be able to measure exposure using outlet density or sales volume instead of an indicator variable.

While mechanisms linking physical availability to consumption-related harms are established for alcohol, it is not yet clear whether the same is true for cannabis.^2^ Storefront recreational cannabis outlets are comparatively rare, and consumers may travel further, purchase cannabis in the illicit market, order home delivery, or otherwise have different patterns of access for cannabis compared with alcohol.^57^ Additionally, people might be consuming other types of cannabinoids that produce effects similar to the delta-9 THC products found in legal outlets, such as hemp-derived delta-8 or delta-10 THC products, which were made available by the 2018 Farm Bill and are now widely accessible.^58,59^

Our finding linking alcohol outlet density to self-harm injuries is consistent with a small body of existing research. Markowitz and colleagues^60^ found that suicide rates are higher in places with higher alcohol outlet density, particularly for men. Similar associations were found in studies in New Mexico, California, and for people aged 15–19 years.^31,61,62^ Alcohol outlet density is recognized to influence alcohol consumption patterns, including risky forms of consumption such as binge drinking, which is also linked to suicidality.^63,64^ Collectively, the research to date supports a role of alcohol outlet density in self-harm and suicide.

If our estimates can be interpreted as causal effects, the subgroup results suggest that higher densities of alcohol outlets may contribute to nonfatal self-harm risk, particularly among women, teenagers and young adults, and Hispanic and White people, some of the groups at highest risk of nonfatal self-harm injury, and to increased suicide risk for men and White people.^32^ Conversely, the results could suggest that local policy changes such as zoning restrictions and caps on alcohol outlet density could especially benefit these groups and thereby reduce disparities. This finding is consistent with public health recommendations to reduce alcohol outlet density as a means of reducing alcohol-related harm.^2,30,65^

### Strengths and Limitations

This study leveraged a large, statewide census of self-harm injuries, captured substantial spatial and temporal variations in cannabis and alcohol outlet densities, assessed heterogeneous treatment effects by population subgroup, and incorporated a novel application g-computation to convert estimates from multiplicative Bayesian spatiotemporal models to additive-scale policy-relevant parameters.

Our study, like all observational studies, has some limitations that could explain our findings due to uncontrolled confounding. First, we were unable to access certain covariates relevant to self-harm at the ZCTA level, such as firearm ownership rates and mental health services utilization. Second, our data did not include all cases of self-harm, as death and hospital discharge data only capture the most serious self-harm injuries. Additionally, we used residential ZIP codes to construct the outcome variable, but self-harm injuries may occur at locations different from the place of residence. This limitation also extends to our inability to examine the mechanisms by which cannabis outlets might affect self-harm rates.

Similarly, we cannot measure the change in self-harm rates in the places that introduced new cannabis outlets: our main analysis does not qualify as a before–after analysis. Another limitation is that we did not capture all the ways in which people can access cannabis, such as through illegal markets, delta-8 products, or delivery services.

Finally, although our spatiotemporal analysis approach accounts for spatial autocorrelation in the outcomes and covariates, we did not consider spatially lagged effects that would capture the impact of cannabis outlets in one ZCTA on rates of self-harm in neighboring ZCTAs. This is particularly relevant considering that instances of recreational cannabis use are comparatively rare and may have spillover effects.“

## CONCLUSIONS

To our knowledge, this is the first study to assess the interaction between cannabis outlets and alcohol outlets in their association with self-harm injuries. Although alcohol outlet density was associated with self-harm, particularly for high-risk population subgroups, cannabis outlets were not, and we found no evidence of interaction. These associations should be examined in different settings. This study adds to the literature on the links between recreational cannabis legalization and commercialization on diverse public health outcomes which can be used to inform decision-making in cannabis and alcohol policy and regulation.

## Supplementary Material
