# Supplementary material for: Examining the Interactive Associations of Cannabis and Alcohol Outlets With Self-harm Injuries in California: A Spatiotemporal Analysis
Source: Epidemiology. 2024 Dec 16;36(2):196–206. doi: 10.1097/EDE.0000000000001822 (PMC11774197; doi:10.1097/EDE.0000000000001822)
Supplement: Supplementary file 1 [file ede-36-196-s001.pdf]

Supplemental Digital Content
Supplemental methods

Cannabis outlet measurement

This study focused on both legal and illegal cannabis outlets in California, as both may affect the availability of cannabis and influence cannabis-related health outcomes. Illegal outlets have historically been prevalent in California, making up as much as 60% of all outlets, and their presence reflects the legacy of the medical cannabis era when regulation was undeveloped and many medical outlets operated in a legal grey space.(1,2) It also reflects that following legalization of adult-use or recreational cannabis in California, existing medical dispensary owners were given the opportunity to convert to recreational outlets. Illegal medical dispensaries and those operating in a legal grey space were also given the opportunity to come into compliance with the new laws, and if they did not do so within the designated timeline, they were subject to abatement. Grandfathered outlets also exist in cities and counties that previous permitted but now ban outlets.

Data on storefront recreational cannabis outlets (hereafter, "outlets") was webscraped annually between 2017 and 2019 from Weedmaps, a high-traffic online promotional cannabis business finder widely used in cannabis research.(3–6) A recent validation study of all storefront cannabis outlets in California found that compared to official license listings or other finders, Weedmaps was the most up-to-date and comprehensive source for capturing both legal and illegal outlets.(1)

We focused on recreational cannabis outlets, as opposed to medical outlets, because following recreational legalization, few medical-only outlets remained. Recreational outlets include both existing retailers that converted from medical to recreational with legalization as well as newly opened retailers. The Weedmaps data did not allow us to distinguish these two types of outlets and thus to examine "churning" of outlets.(7) In our analysis, we treated newly opened recreational outlets as identical to converted medical outlets, but the effects of new recreational outlets may differ from those of converted medical outlets,(7) and this should be examined in future research.

We focused on storefront outlets (also known as brick-and-mortar outlets), as opposed to home delivery retailers, because our study builds on conceptual models and analytic approaches based on physical proximity to outlets where purchases can be made in-person,(8) whereas conceptual models and methods for measuring access to delivery remain undeveloped.(9) In Weedmaps, the majority of delivery-only businesses do not report an address, further justifying this study's focus on storefront outlets. If an outlet offered both a storefront and home delivery, we included it in the count of storefront outlets.

All sources of cannabis outlet data have strengths and limitations.(1,10) Research suggests that online finders like Weedmaps tend to be more up-to-date—better indicating which outlets are newly opened or no longer operating—and are more comprehensive in capturing illegal outlets.(1,2) However, Weedmaps and other online finders are commercial, promotional websites. They are not designed for public health research and are not optimized for generating comprehensive listings of the locations of outlets. The gold standard for generating cannabis outlets listings is direct observation, but this is a time- and cost-intensive endeavor, especially for research that seeks to track changes in outlets over time. California's state cannabis control agency offers official license listings, but such records exclude most illegal outlets, are not updated as frequently as online finders, and sometimes lack the premise addresses needed to identify outlet locations. Merging Weedmaps data with official license listings for California to determine which outlets may be legal or illegal is also challenging and time-intensive because no clear, clean variables exist on which to merge the two sources. None of the available sources keep historical records of outlet listings and therefore listings must be collected regularly and prospectively to construct panel data on outlets over time. It is also possible to

identify illegal outlets is through direct physical observation or using a google street view classification algorithm in comparison with official license listings. Unfortunately, this was not feasible within the timeline or level available through the grant supporting this project.

Identification of self-harm injuries

We acquired statewide records of emergency department (ED) and inpatient hospital discharge (PDD) records from the California Department of Health Care Access and Information, and death records from the California Department of Public Health – Vital Records.

eTable 1: ICD codes used to identify self-harm incidents

	ICD-10-CM codes for ED and PDD	ICD-10 codes for death records
Fatal self-harm	-	X60 – X84, U03
Nonfatal self-harm	X71-X83, T36-T65, T71 (w/ a “2” in the 5th or 6th digit to indicate sdv)	-

Deaths were dropped from hospital and ED data to avoid double-counting. We did not consider alcohol or cannabis consumption of the injured person because death, ED, and hospitalization data provide unreliable information on alcohol and cannabis’s role in violent events (12).

Crosswalk from postal ZIP codes to ZCTAs

ZIP Code Census Tabulation Areas (ZCTAs) are administrative boundaries defined by the US Census Bureau and updated every 10 years with the decennial census. ZCTAs are areal representations of the ZIP code delivery routes developed by the US postal service. We downloaded polygon maps of ZCTAs from the ACS. To crosswalk counts of self-harm injuries from postal ZIP codes to ZCTAs, we used Uniform Data System (UDS) mapper crosswalk (https://udsmapper.org/zip-code-to-zcta-crosswalk/). This website hosts a yearly ZIP code to ZCTA crosswalk from 2009 to 2021, because ZIP codes are updated every year. We wrote an R script to aggregate the outcome data to the ZIP code-quarter level, and merge these data with the UDS crosswalks to determine the ZCTA equivalent for each ZIP code (one-to-one correspondence). UDS mapper is a website developed by the Health Resources and Services Administration, John Snow, Inc. and the American Academy of Family Physicians to inform the geographical development of the U.S. Federal Health Center Program.

Potential confounders
eTable 2 provides detail on the data sources and procedures for each covariate. We conceptualized race/ethnicity as socially-defined categories that reflect the distribution of risk, opportunities, and discrimination.(13)

eTable 2: Potential confounder data sources and measures

**Measures**	**Data source**	**Notes**
Demographics covariates: Population density, median age, % non-Hispanic Black alone, % Hispanic or Latinx, % non-Hispanic Asian alone, % renters, % veterans<br>Socioeconomic covariates: educational attainment, median income, poverty, unemployment, average household size, Gini coefficient, % families in poverty, % with high school degree, % with some education after high school, % with some degree after high school, % with at least a Bachelor's degree, % with advanced degree, % living below 150% of the federal poverty level	American Community Survey, 2017-2019, ZCTA level	We ZCTA-level estimates corresponding to 5-year averages ending in the designated study year. For example, estimates for 2017 used the 2013-2017 ACS files.<br><br>We did not consider median income because missingness for this variable was unusually high: 279 unique ZCTAs were missing median income.
% of suicide deaths completed with a firearm	California death records	This is a proxy for firearm access.
Overall rate of ED visits and inpatient hospitalizations	California ED and hospital discharge records	This is a proxy for overall healthcare utilization.
Densities of retail outlets (all types), social organizations, services (all types, e.g., food services), tobacco outlets, payday loan outlets, and pawn shops, per 100,000 people	US Census Zip Codes Business Patterns data	These variables were ultimately excluded because this data series was discontinued in 2018 and thus was completely missing for study year 2019.

Supplemental results

eTable 3: Estimated hyperparameters in fully adjusted spatiotemporal models evaluating the associations of alcohol and cannabis outlet densities with self-harm injuries, California, 2017-2019.

**Outcome**	**Hyperparameter**	Estimate (95% CI)
Nonfatal Self-harm	Marginal precision of BYM2 random effects	5.71 (4.57, 7.08)
	Proportion of marginal variance explained by BMY2 spatial effect (versus i.i.d. effect)	0.68 (0.56, 0.78)
Fatal self-harm	Marginal precision of BYM2 random effects	26.29 (16.9, 39.8)
	Proportion of marginal variance explained by BMY2 spatial effect (versus i.i.d. effect)	0.62 (0.33, 0.86)

eTable 4: Estimated Watanabe-Akaike Information Criterion (WAIC) for main models and secondary and sensitivity analyses

Outcome means	Sensitivity Test	WAIC	Improved model fit
Nonfatal Self-harm	Main model	76594.66	N/A
	Quadratic AOD term added	76597.39	No
	ZCTA fixed effects added	105266.69	No
	Terms for % bar/pub and % off-premise added	76597.71	No
	Spatially lagged AOD exposure term added	76597.99	No
	Spatially lagged cannabis outlet exposure term added	76598.29	No
	Baseline medical dispensary density term added	76591.05	No
	Fatal and nonfatal self-harm outcomes	78373.67	No
	Excluded ZCTAs with populations less than 500	75101.04	Yes
	ZCTA-level random slopes added	76157.72	Yes
Fatal self-harm	Main model	32672.11	N/A
	Quadratic AOD term added	32672.31	No
	ZCTA fixed effects added	37755.04	No
	Terms for % bar/pub and % off-premise added	32677.78	No
	Spatially lagged AOD exposure term added	32675.34	No
	Spatially lagged cannabis outlet exposure term added	32674.71	No
	Baseline medical dispensary density term added	32671.35	No
	ZCTA-level random slopes added	32673.44	No
	Excluded ZCTAs with populations less than 500	32284.28	Yes

Legend: A lower WAIC value indicates better model fit. Consistent with prior research, we considered a 5-unit reduction in the Watanabe-Akaike Information Criterion to indicate improved model fit.(14,15)

Note: Models with ZCTA fixed effects theoretically provided stronger confounder control, and results from these models were in the same direction as the main findings, but estimates were unstable and imprecise.

eTable 5: Estimated risk ratios for main models and sensitivity analyses that improved model fit

**Outcome means**	**Model**	**Variable**	**RR [95% CI]**
Nonfatal self-harm	Primary analysis	Alcohol outlets per 100,000 (AOD)	1.0001 [1.0001, 1.0002]
		Any cannabis outlets	0.9957 [0.9615, 1.0312]
		{AOD}*{Any cannabis outlets}	0.9999 [0.9989, 1.0000]
	Sensitivity Analysis: Fatal and non-fatal self-harm incidents combined	Alcohol outlets per 100,000 (AOD)	1.0002 [1.0001, 1.0002]
		Any cannabis outlets	0.9979 [0.9649, 1.0320]
		{AOD}*{Any cannabis outlets}	0.9999 [0.9999, 1.0000]
	Sensitivity Analysis: Excluding ZCTAS with a population of 500 or less	Alcohol outlets per 100,000 (AOD)	1.0002 [0.9967, 1.0911]
		Any cannabis outlets	0.9990 [1.0002, 1.0003]
		{AOD}*{Any cannabis outlets}	0.9999 [0.9998, 1.0000]
	Sensitivity Analysis: ZCTA-level Random Slopes	Alcohol outlets per 100,000 (AOD)	1.0001 [1.0001, 1.0002]
		Any cannabis outlets	1.0077 [0.9672, 1.0501]
		{AOD}*{Any cannabis outlets}	0.9999 [0.9986, 1.0000]
	Rural only ZCTAs	Alcohol outlets per 100,000 (AOD)	1.0000 [0.9998, 1.0001]
		Any cannabis outlets	0.9844 [0.8035, 1.2076]
		{AOD}*{Any cannabis outlets}	1.0000 [0.9994, 1.0005]
	Urban only ZCTAs	Alcohol outlets per 100,000 (AOD)	1.0002 [1.0002, 1.0003]
		Any cannabis outlets	1.0019 [0.9672, 1.0379]
		{AOD}*{Any cannabis outlets}	0.9999 [0.9998, 1.0000]
Fatal self-harm	Primary Analysis	Alcohol outlets per 100,000 (AOD)	1.0001 [1.2638, 1.3661]
		Any cannabis outlets	0.9706 [0.8787, 1.0721]
		{AOD}*{Any cannabis outlets}	1.0002 [0.9999, 1.0004]
	Sensitivity Analysis: Excluding ZCTAS with a population of 500 or less	Alcohol outlets per 100,000 (AOD)	1.0001 [1.0000, 1.0002]
		Any cannabis outlets	0.9718 [0.8794, 1.0739]
		{AOD}*{Any cannabis outlets}	1.0002 [0.9999, 1.0004]
	Rural only ZCTAs	Alcohol outlets per 100,000 (AOD)	1.0000 [0.9997, 1.0002]
		Any cannabis outlets	0.7724 [0.4504, 1.3280]
		{AOD}*{Any cannabis outlets}	1.0005 [0.9992, 1.0017]
	Urban only ZCTAs	Alcohol outlets per 100,000 (AOD)	1.0001 [1.0001, 1.0002]
		Any cannabis outlets	0.9779 [0.8839, 1.0819]
		{AOD}*{Any cannabis outlets}	1.0002 [0.9999, 1.0004]

eTable 6: Average self-harm rates through the period of study

Legend: Mean and standard deviation self-harm rates in the state of California for the ZCTAs in the study.

**Self-harm rate per 100,000 per quarter Mean (standard deviation)**		
Year	**Fatal**	**Nonfatal**
2017	5.75 (114.92)	37.15 (80.66)
2018	4.44 (32.29)	38.35 (107.03)
2019	4.02 (24.64)	35.48 (75.7)

Rural Urban Secondary Analysis

eFigure 1: **Adjusted risk difference in nonfatal self-harm injuries under hypothetical reductions in alcohol and cannabis outlet densities, California, 2017-2019 in urban only ZCTAs**
Legend: Estimated differences in the risk of nonfatal self-harm injuries corresponding to hypothetical reductions in alcohol and cannabis outlet densities, calculated using the posterior distributions of the fitted models. We estimated the change in nonfatal self-harm risk if storefront recreational cannabis outlets had never opened (i.e., setting cannabis outlets to 0 throughout) if alcohol outlet density were X% lower in each ZCTA-quarter where X ranged from 10% to 90%, and if both shifts occurred simultaneously. Estimates are reported as marginal posterior median risk differences per 100,000 with 95% credible intervals.

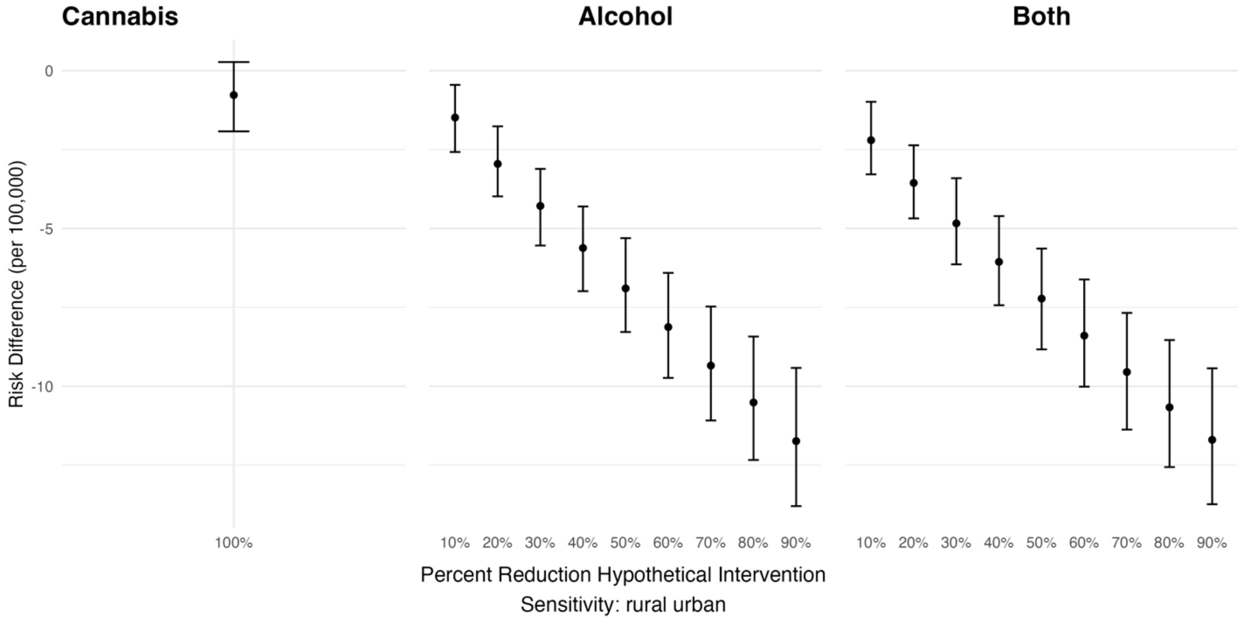

eFigure 2: **Adjusted risk difference in fatal self-harm injuries under hypothetical reductions in alcohol and cannabis outlet densities, California, 2017-2019 in urban only ZCTAs**
Legend: Estimated differences in the risk of fatal self-harm injuries corresponding to hypothetical reductions in alcohol and cannabis outlet densities, calculated using the posterior distributions of the fitted models. We estimated the change in nonfatal self-harm risk if storefront recreational cannabis outlets had never opened (i.e., setting cannabis outlets to 0 throughout) if alcohol outlet density were X% lower in each ZCTA-quarter where X ranged from 10% to 90%, and if both shifts occurred simultaneously. Estimates are reported as marginal posterior median risk differences per 100,000 with 95% credible intervals.

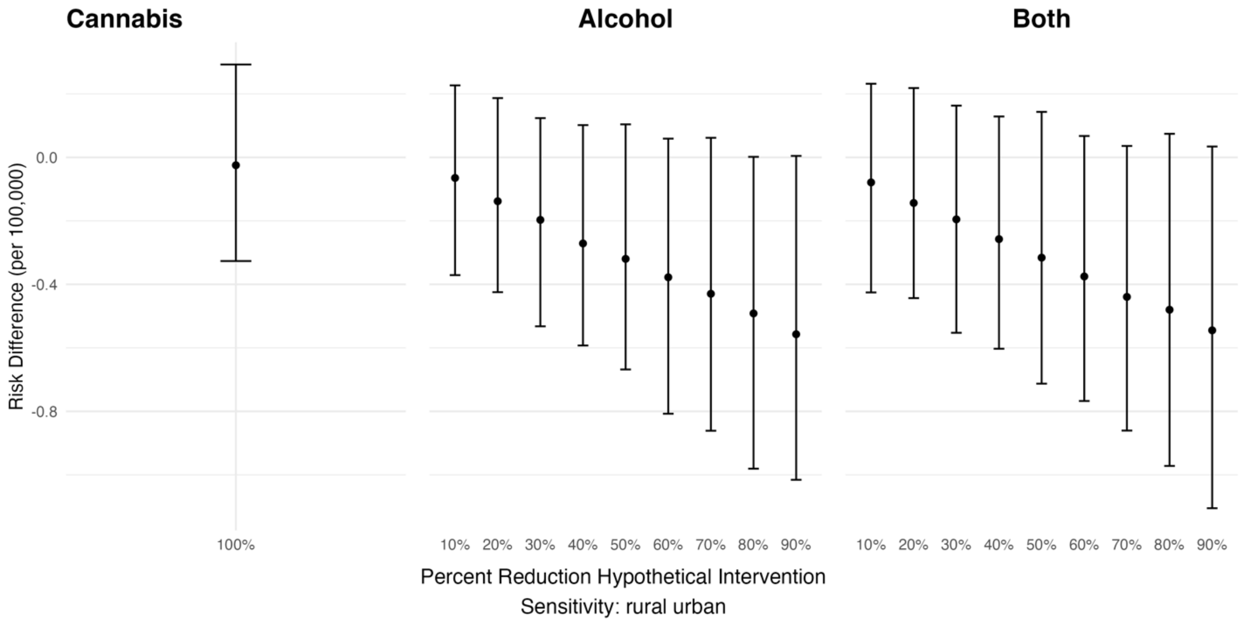

eFigure 3: **Adjusted risk difference in nonfatal self-harm injuries under hypothetical reductions in alcohol and cannabis outlet densities, California, 2017-2019 in rural only ZCTAs**

Legend: Estimated differences in the risk of nonfatal self-harm injuries corresponding to hypothetical reductions in alcohol and cannabis outlet densities, calculated using the posterior distributions of the fitted models. We estimated the change in nonfatal self-harm risk if storefront recreational cannabis outlets had never opened (i.e., setting cannabis outlets to 0 throughout) if alcohol outlet density were X% lower in each ZCTA-quarter where X ranged from 10% to 90%, and if both shifts occurred simultaneously. Estimates are reported as marginal posterior median risk differences per 100,000 with 95% credible intervals.

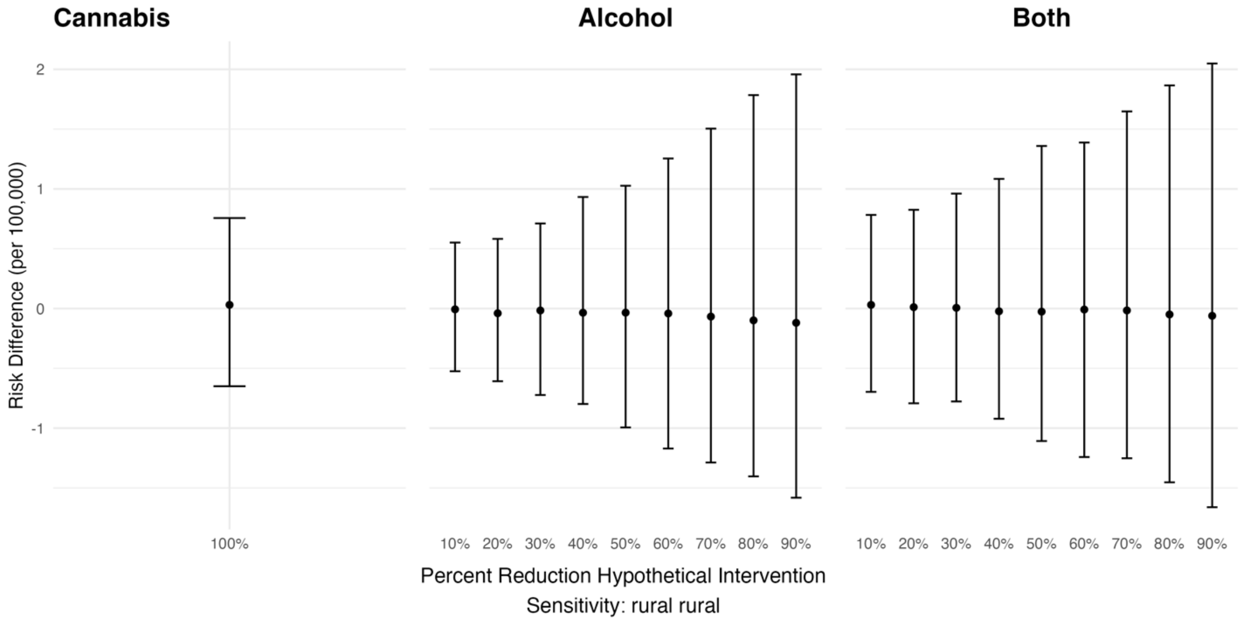

Self-harm Rate Maps

eFigure 4: **Aggregated fatal self-harm rates in California at the ZIP code census tabulation areas (ZCTA) 2017 through 2019.**

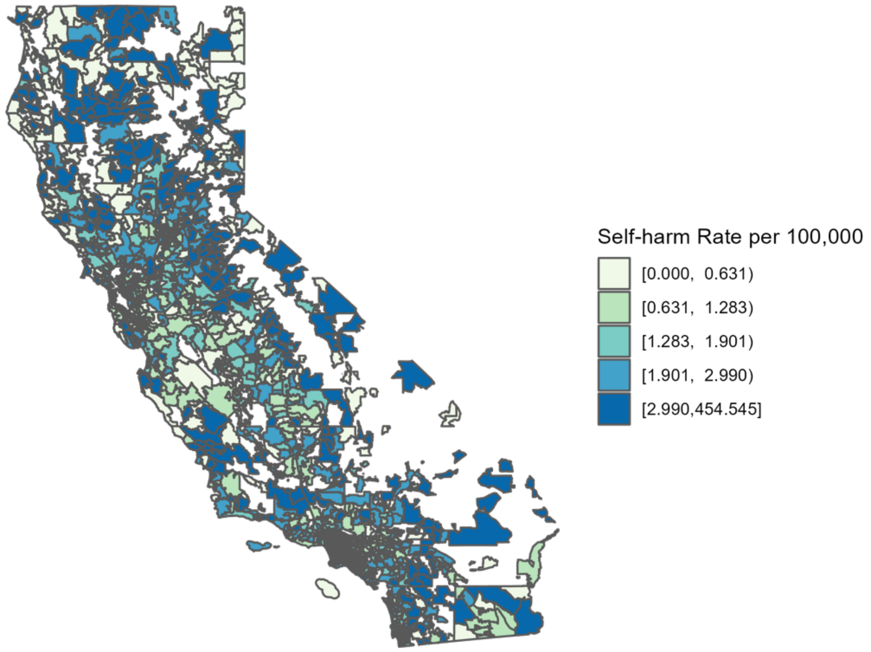

eFigure 5: **Aggregated non-fatal self-harm rates at the ZIP code census tabulation areas (ZCTA) 2017 through 2019.**

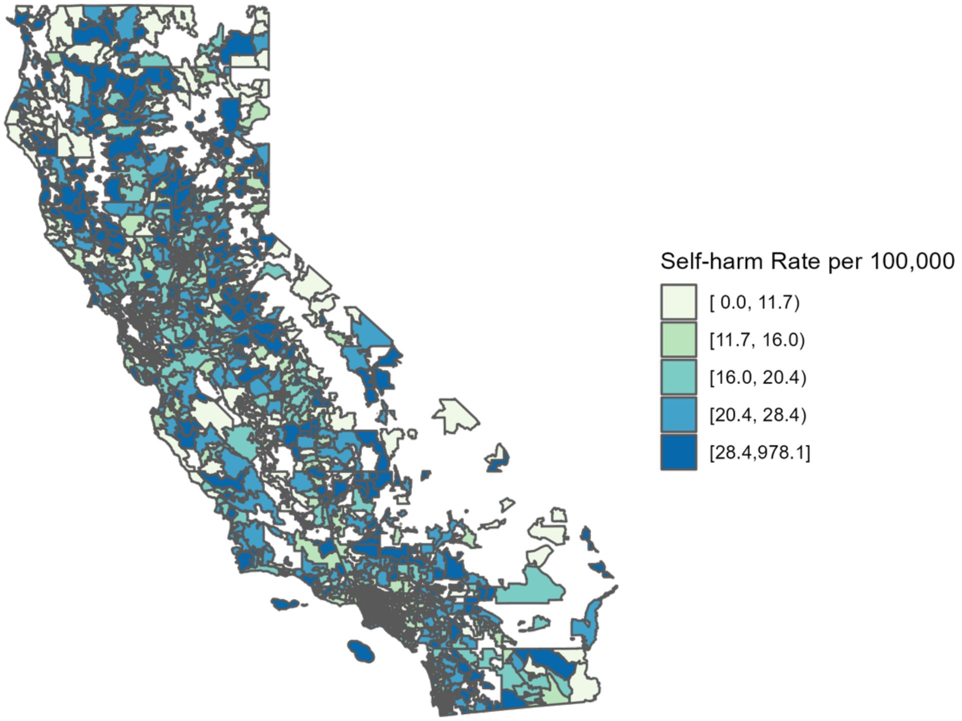
Self-harm Rate per 100,000
[0.0, 11.7)
[11.7, 16.0)
[16.0, 20.4)
[20.4, 28.4)
[28.4,978.1]

R statistical code to implement estimation

1 Set up packages, bring in and merge data, define expected outcomes

```
# Packages
library('INLA')
library('sf')
library('tidyverse')
library('tidycensus')
library('spdep')
library('knitr')
library('janitor')


# Settings

fatal <- "nonfatal" # fatal, nonfatal, both
outcome <- "selfharm" #chr: selfharm, assault
agegrp <- "total" # "total" = all age groups  num:, 1 = [0 - 14], 2 == [15- 34], 3 ==
[35 - 54], 4 == 55+
sex <- "total" #chr: total, male, female
race <- "total" # chr: total; white, black, amer_indian, asian_pac, two_more, hisp
means <- "nonfirearm" # chr: Total; firearm; nonfirearm.

# So that random processes are reproducible.
set.seed(1000)

# Load Predictor data
load("./data/predictor_data_complete.RData")

# Load Outcome Data
dta <- read.csv(paste0("./data/data17-19_crosswalk_outcome_", outcome, "_fatal_status
_",
                 fatal,"_means_", means,"_sex_", sex,
                 "_race_", race,"_agegrp_", agegrp, ".csv"))

dta_full <- right_join(dta, X,
                  by=c('zcta','year','quarter','t')
                  ) |>
          # Create the rate using the data that comes from
         # the predictor data
         mutate(
           #If the number of cases is NA, give it a 0
                n = ifelse(is.na(n), 0, n),
                # for the zctas that do not appear in the predictor or outcome
                num_visits = ifelse(is.na(num_visits), 0, num_visits),
          # create a rate for the hospital visits
          num_visits_pc = num_visits/pop_total_total_total*100000
                )

# Define the Expected Outcomes
dta_full$y <- dta_full$n

rate <- sum(dta_full$n) / sum(dta_full$total_pop) # Calculate the state-year-wide rat
e of cases per population (rates are in quarterly units)
dta_full$E <- dta_full$total_pop * rate
```

2 Setting up the spatial component

```
## Read zcta maps
map <- st_read("./data/tl_2010_06_zcta510/tl_2010_06_zcta510.shp")

# Make sure map and data are ordered the same way
dta_full <- dta_full[order(dta_full$zcta, dta_full$t),]
map <- map[order(map$ZCTA5CE10),]

# restrict the maps to the zctas that are actually in the data
map <- map[map$ZCTA5CE10 %in% unique(dta_full$zcta), ]

# Specify the spatial relationships
temp <- poly2nb(map)
nb2INLA("./data/CA.graph", temp) # saves file to working directory
CA.adj <- paste0(getwd(),"/data/CA.graph") # pull the file
H <- inla.read.graph(filename = "./data/CA.graph") # to import the graph in t
he R format

# Adjacency matrix
adj <- inla.graph2matrix(H)

# Make a sequential ID for ZCTA
dta_full$sequential_ID <- rep(1:Nareas, each = Ntimes) # This is the original
# Make a sequential time variable
dta_full$time_ID <- rep(1:Ntimes, Nareas) # This is the original

# Main effect for time should be factor
dta_full$t <- as.factor(dta_full$t)
```

3 INLA Model

```
my_formula <- "y ~ total_alcohol_pc*disp_nomed_bin + t +
              f(sequential_ID, model='bym2', graph=CA.adj, adjust.for.con.com
p=T, scale.model=T) +
p_pop_under15_sd + p_pop15to34_sd + avg_household_size_sd + p_black_sd + p_ve
teran_sd + p_post_bachelors_sd"


myinla <- inla(my_formula,
                  family='poisson',
                  data=dta_full,
                  E=E,
                  verbose=F,
                  control.predictor=
                  list(compute=T), control.compute=list(config = TRUE, dic=T,
waic=T,cpo=T))
```

4 Risk Difference

```
#' This function operates on the posterior and calculates the PAR parameter f
or a 100% reduction in alcohol outlets
#' @args N Number of samples I want from the poisson
#' @args E Expected number of cases
#' @args x1 Vector from the data set representing the actual values of the di
sp_nomed_bin variable
#' @args x2 Vector from the data set representing the actual values of the to
tal_alcohol_pc variable
#' @args pop is the population of each zcta-quarter
asis_vs_without <- function(N, x1, x2, E, pop) {

  #this function compares the as_is cases vs everyone without cases
  y.as_is <- rpois(N, E * exp(Predictor))
  y.without <- rpois(N, E * exp(Predictor - disp_nomed_bin * x1 -
                                 x1 * x2 * total_alcohol_pc:disp_nomed_bin))
  return((sum(y.without) - sum(y.as_is)) / sum(pop) * 100000)
}

#' This function calculates the PAR parameter for a percentage change in alco
hol outlets
#' @args N Number of samples I want from the poisson
#' @args E Expected number of cases
#' @args x1 Vector from the data set representing the actual values of the di
sp_nomed_bin variable
#' @args x2 Vector from the data set representing the actual values of the to
tal_alcohol_pc variable
#' @args per_red represents a reduction of X% in the value o the x1 variable
(total_alcohol_pc)
#' @args pop is the population of each zcta-quarter
alc_percentage_red_par <- function(N, x1, x2, E, per_red, pop) {
    # reduction in alcohol only

    # This function compares as is vs. a X per reduction relative to the curr
ent value
    y.as_is <- rpois(N, E * exp(Predictor))
    y.perc_less <- rpois(N, E * exp(Predictor -
                                 total_alcohol_pc * x1 +
                                 (1 - per_red) * total_alcohol_pc * x1 -
                                 x1 * x2 * total_alcohol_pc:disp_nomed_bin +
                  (1 - per_red) * x1 * x2 * total_alcohol_pc:disp_nomed_bin
    ))

    return((sum(y.perc_less) - sum(y.as_is)) / sum(pop) * 100000)
  }
#' This function applies the function alc_percentage_red_par to different per
centages
#' @args x a value between 0 to 1 representing the percentage reduction in to
tal_alcohol_pc
```

```
 myinla.eval.par <- function(x) {
    return(inla.posterior.sample.eval(
      alc_percentage_red_par,
      posterior,
      N = nrow(dta_full),
      per_red = x,
      E = myinla[['.args']][['E']],
      x1 = dta_full$total_alcohol_pc,
      x2 = dta_full$disp_nomed_bin,
      pop = pop)
    )
  }

#' This function calculates the PAR parameter for a percent reduction in alco
hol outlets and a 100% reduction
#' in cannabis outlets
#' @args N Number of samples I want from the poisson
#' @args E Expected number of cases
#' @args x1 Vector from the data set representing the actual values of the di
sp_nomed_bin variable
#' @args x2 Vector from the data set representing the actual values of the to
tal_alcohol_pc variable
#' @args per_red represents a reduction of X% in the value o the x2 variable
(disp_nomed_bin)
#' @args pop is the population of each zcta-quarter
alc_percentage_inter_par <- function(N, x1, x2, E, per_red, pop) {
    # reduction in alcohol and no medical dispensaries
    # This function compares as is vs. a X per reduction relative to the curr
ent value
    y.as_is <- rpois(N, E * exp(Predictor))
    y.perc_less <- rpois(N, E * exp(Predictor -
                                    total_alcohol_pc * x1 +
                                 (1 - per_red) * total_alcohol_pc * x1 -
                                 x2 * disp_nomed_bin -
                                 x1 * x2 * total_alcohol_pc:disp_nomed_bin))

    return((sum(y.perc_less) - sum(y.as_is)) / sum(pop) * 100000)
  }

#'This functions calculates the alc_percentage_inter_par reduction for an X%
reduction in total_alcohol_pc
#' @args x a value between 0 to 1 representing the percentage reduction in to
tal_alcohol_pc
  myinla.eval.par.inter <- function(x) {
    return(inla.posterior.sample.eval(
      alc_percentage_inter_par,
      posterior,
      N = nrow(dta_full),
      per_red = x,
      E = myinla[['.args']][['E']],
```

```
      x1 = dta_full$total_alcohol_pc,
      x2 = dta_full$disp_nomed_bin,
      pop = pop
    )
    )
  }

# Sample from the posterior
posterior <- inla.posterior.sample(1000, myinla, seed = 1234)

# Apply asis_vs_without function to the posterior
asis_vs_without_eval <- inla.posterior.sample.eval(asis_vs_without,
                                                   posterior,
                                                   ## pass variables
                                             N = nrow(dta_full),
                                             E = myinla[[".args"]][["E"]],
                                             x1 = dta_full$disp_nomed_bin,
                                             x2 = dta_full$total_alcohol_pc,
                                             pop = pop
)

# Apply function alc_percentage_red_par to the posterior with different perce
ntage reductions
myinla.eval.par.lapply = mclapply(seq(0.1, 0.9, 0.1), myinla.eval.par, mc.cor
es = 3)

# Apply function alc_percentage_inter_par to the posterior with different per
centage reductions
myinla.eval.par.inter.apply = mclapply(seq(0.1, 0.9, 0.1), myinla.eval.par.in
ter, mc.cores = 3)
```

REFERENCES

1. Cao Y, Carrillo AS, Jankowska MM, Shi Y. Validation of secondary data sources for enumerating marijuana dispensaries in a state commercializing marijuana. Drug and Alcohol Dependence. 2020 Oct 1;215:108183.

2. Unger JB, Vos RO, Wu JS, Hardaway K, Sarain AYL, Soto DW, et al. Locations of licensed and unlicensed cannabis retailers in California: A threat to health equity? Prev Med Rep [Internet]. 2020 Jul 13 [cited 2020 Aug 31];19. Available from: https://www.ncbi.nlm.nih.gov/pmc/articles/PMC7378688/

3. Freisthler B, Gruenewald PJ. Examining the relationship between the physical availability of medical marijuana and marijuana use across fifty California cities. Drug and Alcohol Dependence. 2014 Oct 1;143:244–50.

4. Shi Y, Meseck K, Jankowska MM. Journal of Addiction. 2016 [cited 2019 Apr 4]. Availability of Medical and Recreational Marijuana Stores and Neighborhood Characteristics in Colorado. Available from: https://www.hindawi.com/journals/jad/2016/7193740/abs/

5. Lipperman-Kreda S, Lee JP, Morrison C, Freisthler B. Availability of tobacco products associated with use of marijuana cigars (blunts). Drug and Alcohol Dependence. 2014 Jan 1;134:337–42.

6. Mair C, Freisthler B, Ponicki WR, Gaidus A. The impacts of marijuana dispensary density and neighborhood ecology on marijuana abuse and dependence. Drug and Alcohol Dependence. 2015 Sep 1;154:111–6.

7. Minner JS, Shi X. Churn and change along commercial strips: Spatial analysis of patterns in remodelling activity and landscapes of local business. Urban Studies. 2017 Dec 1;54(16):3655–80.

8. Gruenewald PJ. Regulating availability: how access to alcohol affects drinking and problems in youth and adults. Alcohol Res Health. 2011;34(2):248–56.

9. Matthay EC, Schmidt LA. Home delivery of legal intoxicants in the age of COVID-19. Addiction. 2020 Oct 13;

10. Pedersen ER, Firth C, Parker J, Shih RA, Davenport S, Rodriguez A, et al. Locating Medical and Recreational Cannabis Outlets for Research Purposes: Online Methods and Observational Study. J Med Internet Res [Internet]. 2020 Feb 26 [cited 2020 Aug 31];22(2). Available from: https://www.ncbi.nlm.nih.gov/pmc/articles/PMC7066509/

11. Matthay EC, Farkas K, Goin DE, Rudolph KE, Pear VA, Ahern J. Associations of firearm dealer openings with firearm self-harm deaths and injuries: A differences-in-differences analysis. PLOS ONE. 2021 Mar 18;16(3):e0248130.

12. Ye Y, Shield K, Cherpitel CJ, Manthey J, Korcha R, Rehm J. Estimating alcohol-attributable fractions for injuries based on data from emergency department and observational studies: a comparison of two methods. Addiction. 2019 Mar;114(3):462–70.

13. Jones CP. Invited Commentary: “Race,” Racism, and the Practice of Epidemiology. American Journal of Epidemiology. 2001 Aug 15;154(4):299–304.

14. Watanabe S, Opper M. Asymptotic equivalence of Bayes cross validation and widely applicable information criterion in singular learning theory. Journal of Machine Learning Research. 2010;11(12).

15. Burnham K, Anderson D. Model Selection and Multimodel Inference: A Practical and Information-Theoretic Approach. New York: Springer; 2010.
